# Rethinking Measures of Functional Connectivity via Feature Extraction

**DOI:** 10.1038/s41598-020-57915-w

**Published:** 2020-01-28

**Authors:** Rosaleena Mohanty, William A. Sethares, Veena A. Nair, Vivek Prabhakaran

**Affiliations:** 1 Department of Radiology, University of Wisconsin–Madison, Madison, WI USA; 2 Electrical Engineering, University of Wisconsin-Madison, Madison, WI USA; 3 Department of Psychiatry, University of Wisconsin-Madison, Madison, USA; 4 Department of Medical Physics, University of Wisconsin–Madison, Madison, WI USA; 5 Karolinska Institutet, Huddinge, Sweden

**Keywords:** Network models, Diagnostic markers

## Abstract

Functional magnetic resonance imaging (fMRI)-based functional connectivity (FC) commonly characterizes the functional connections in the brain. Conventional quantification of FC by Pearson's correlation captures linear, time-domain dependencies among blood-oxygen-level-dependent (BOLD) signals. We examined measures to quantify FC by investigating: (i) Is Pearson's correlation sufficient to characterize FC? (ii) Can alternative measures better quantify FC? (iii) What are the implications of using alternative FC measures? FMRI analysis in healthy adult population suggested that: (i) Pearson's correlation cannot comprehensively capture BOLD inter-dependencies. (ii) Eight alternative FC measures were similarly consistent between task and resting-state fMRI, improved age-based classification and provided better association with behavioral outcomes. (iii) Formulated hypotheses were: first, in lieu of Pearson’s correlation, an augmented, composite and multi-metric definition of FC is more appropriate; second, canonical large-scale brain networks may depend on the chosen FC measure. A thorough notion of FC promises better understanding of variations within a given population.

## Introduction

Characterization of the brain connectome has been highlighted by numerous human and animal studies^1–3^ which have provided useful and meaningful information to explain a wide range of pathological conditions and behavioral traits in various population groups. The brain connectome can be understood using many measures of connectivity of distinct nature: structural (using imaging techniques such as T1 and diffusion magnetic resonance imaging (MRI)), functional (using functional imaging such as positron emission tomography and functional MRI) as well as neuronal (using scalp recordings such as in magnetoencephalogram (MEG) and electroencephalogram (EEG)).

Functional connectivity (FC), originally defined as the statistical dependencies among neurophysiological events of anatomically distinct brain regions in positron emission imaging^4^, was subsequently applied to functional MRI (fMRI) data^5^. Most conventionally, FC is defined by measuring similarity between brain signals arising from two regions. Under a traditional notion of similarity such as Pearson’s correlation, signals from two anatomically separated brain regions may appear correlated and hence indicate that the regions are functionally connected in the brain^6–9^. It must be noted, however, that a strong correlation between two regions may not guarantee a functional connection of the underlying neurons. For instance, there may exist a reasonable correlation of neuronal activity of two regions under the influence of external or common inputs. Although the concepts of similarity and dissimilarity appear simple, mathematical formulations reveal otherwise. Statistical dependencies between two signals can arise in a number of different ways. Two distinct similarity measures may not measure similarity in the same way. Likewise, a similarity measure may not bear a direct and simple inverse relationship to a dissimilarity measure.

The implication that functional connectivity is dependent upon the measure used to quantify it remains relatively unexplored. Brain regions may appear connected functionally under one definition but disconnected under another^10–16^ irrespective of structural connectivity. The notion of functional connectivity has driven characterization of neural bases not only in the typical healthy population but also various pathological groups demonstrating aberrant patterns, including but not limited to neurological (e.g. stroke, epilepsy)^15,16^, neurodegenerative (e.g. Alzheimer’s disease, dementia)^11,14^ and psychiatric (e.g. depression, schizophrenia)^12,13^ brain diseases. Thus, it would be key to understand the various definitions of connectivity relative to each other in order to sensibly choose a measure to be used, which could, in turn, be utilized to update and/or improve the current knowledge of the brain connectome. A particular definition of connectivity may also convey distinct types of information about the neural bases.

In brief, the goal of this study was to identify multiple alternative measures that could be used to quantify FC. These measures may be complementary to one another and the goal is to assess them by formulating research questions specific and relevant to the study of connectivity in neuroscience. In particular, the following three research questions were formulated: (i) Does Pearson’s correlation, which is the conventional way of defining FC, provide a sufficient characterization of it? (ii) Are there alternative measures that could be used to better quantify FC? (iii) How do the measures of FC compare relatively for population-based classification and prediction of behavioral data? (iv) What are the implications of using varying measures of FC? (v) What could be done to choose the best notion(s) of FC?

In this exploratory study, functional MRI data-based experimentation was adopted in healthy human population to answer these questions. The preliminary results indicated that: (i) the conventional measure of Pearson’s correlation alone may provide an incomplete characterization of FC; (ii) there exist several alternative measures that can capture interactions between brain signals in different ways; (iii) no single measure of FC stands out in the context of classification or prediction; (iv) the idea of large-scale brain connectivity or functional configuration of the brain, largely identified on the basis of Pearson’s correlation, may look different under the chosen definition of FC; and (v) rather than relying on one single measure of FC, a wiser option may be to combine multiple complementary measures of FC, choose a subset of them on the basis of feature selection to avoid overfitting and use the more comprehensive multi-metric definition.

## Results

### Incomplete characterization of functional connectivity

**Figure 1:**
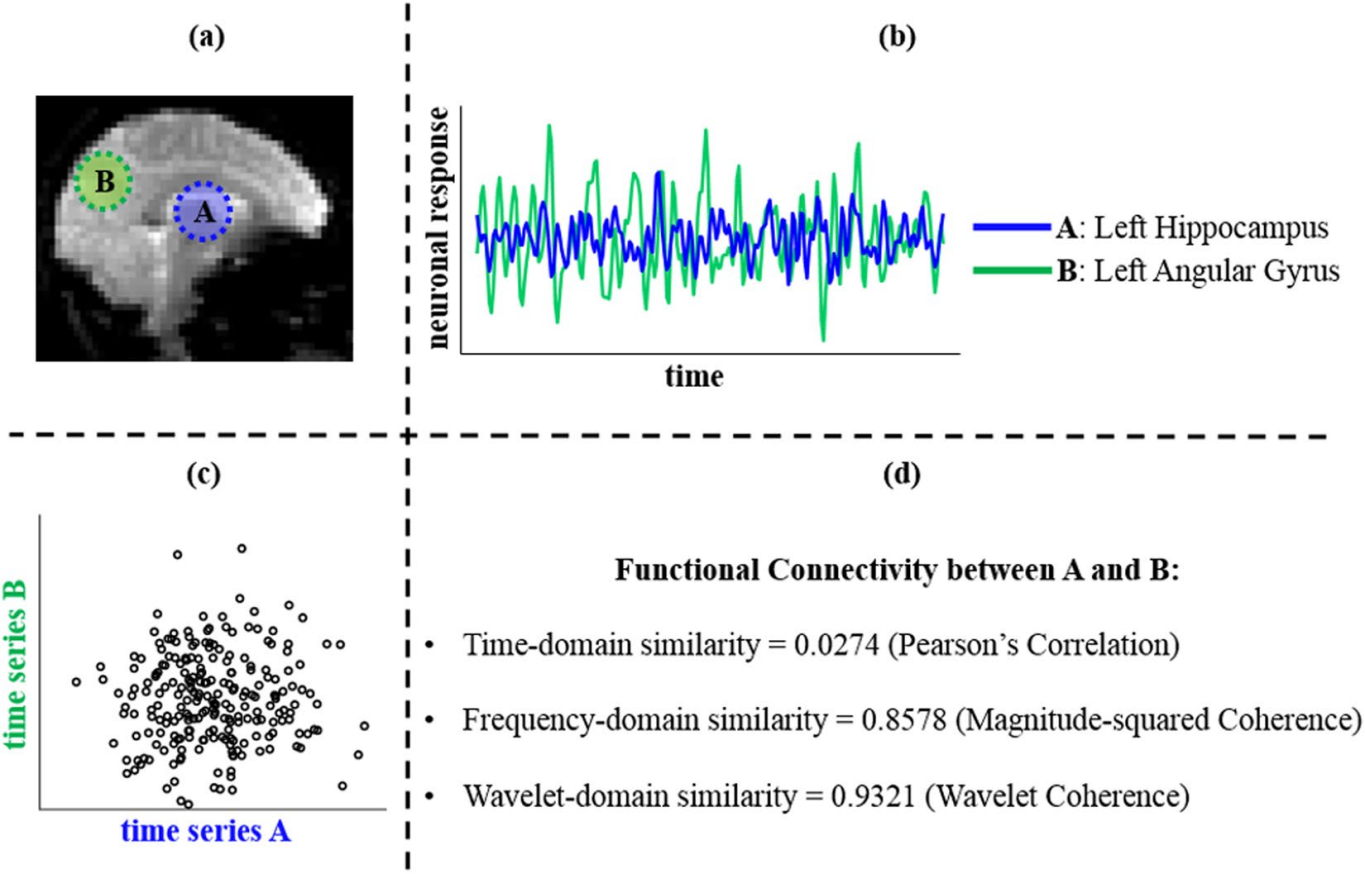
A contrary case. **Note:** An example of FC within the standard DMN in a young healthy adult: (**a**) left hippocampus (denoted by A) and left angular gyrus (denoted by B) within the DMN are considered based on the Willard functional atlas; (**b**) the BOLD time series signals from preprocessed resting-state functional MRI were extracted from each region; (**c**) a scatter plot comparing the two BOLD time series shows the temporal linear correlation between them; (**d**) three distinct similarity measures of FC between the signals are compared; FC = functional connectivity; DMN = default mode network; BOLD = blood-oxygen-level-dependent;.

Cases of the like presented in Fig. 1 were found not to be limited to the default mode network (DMN) alone in healthy participants. Such cases are evidence in support for the claim that FC cannot always be completely quantified by Pearson’s correlation, and especially misses out on individual-level variations. This can also be mathematically substantiated by considering the extreme case of zero Pearson’s correlation between two signals. If two signals are uncorrelated, independence is not necessarily implied. More generally, in cases where a low value of Pearson’s correlation is observed, it may be incorrect to assume that there is no dependence between them. It simply means that there is no linear dependence between them.

### Alternative characterizations of functional connectivity

This study was aimed at investigating alternative ways of characterizing FC that could augment and be complementary to Pearson’s correlation. FC was evaluated with eight alternative measures namely: cross-correlation, coherence, wavelet coherence, mutual information, Euclidean distance, cityblock distance, dynamic time warping and earth mover’s distance. These measures were included as they capture different aspects of statistical dependence between BOLD signals (such as time-, frequency- and wavelet-domain information, similarity and dissimilarity measures, linear and non-linear dependencies). These different measures were compared and contrasted with three experiments which assessed the consistency of information provided by the FC measures, utility of the FC measures in population-based discrimination and the biological plausibility of using these FC measures.

### Experiment 1 (E1)

The goal of the first experiment was to understand the relative consistency of the different FC measures when evaluated in young and healthy individuals. Within-individual consistency was first established by comparing the overlap (Sørensen-Dice similarity coefficient) of the FC pattern observed in task (motor and verbal) and resting-state functional MRI and then averaging across individuals for each FC measure.

### Findings from E1: Consistency of functional connectivity

**Figure 2:**
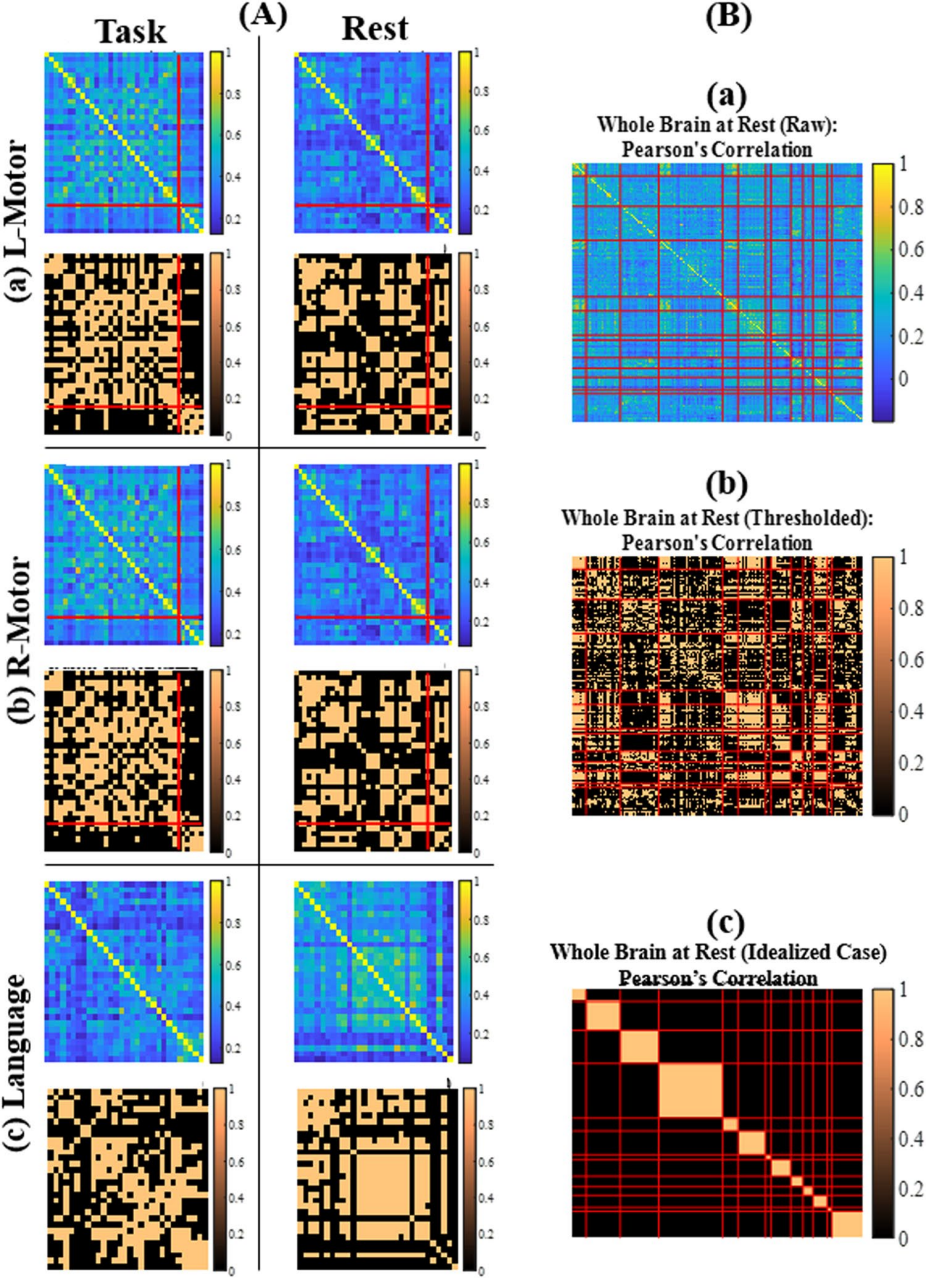
FC in 19 young healthy adults. **(A)** Comparison of FC based on Pearson’s correlation in between task and resting-state conditions for **E1** in: (a) left motor (b) right motor and (c) language networks. **(B**) Whole brain resting-state FC defined based on Pearson’s correlation in 13 distinct brain networks given by Power functional atlas in **E1**. **Note: (A)** defined based on Power functional atlas. In each sub-image, the first row represents the FC matrix averaged across all 19 participants (i.e. each cell was the average of individual FC values in the cell) and the second row represents the thresholded FC matrix averaged across all participants. The red lines in (a) and (b) show the separation between hand-motor and mouth-motor brain regions. Similar matrices for the alternative measures of FC can be found in Supplementary Fig. 2. **(B)** (a) FC matrix averaged across all 19 participants (i.e. each cell was the average of individual FC values in the cell); (b) thresholded FC matrix averaged across all participants; (c) simulated idealized FC matrix. The red lines represent the separation between brain regions belonging to a specific network. The regions are grouped in the following order: audio, visual, motor, default mode, cingulo-opercular task, fronto-parietal task, memory, salience, dorsal attention, ventral attention, subcortical, cerebellar, uncertain networks. Similar matrices for the alternative measures of FC can be found in Supplementary Fig. 3.

Consistency of all of the identified FC measures was evaluated by comparing task functional MRI and resting-state functional MRI in young healthy adults (demographics in Table 1). Since the connectivity pattern may be different for each FC measure, the task functional MRI data offer a form of ground truth as they activate specific regions in the brain which can bear correspondence with those in resting condition. Thresholded FC maps (threshold = one standard deviation above grand mean) between task and resting-state conditions are illustrated for Pearson’s correlation in Fig. 2 (subfigure A) and remaining FC measures in Supplementary Fig. 1.

**Table 1:** Characteristics of young healthy participants included in **E1**.

Characteristic	Value
N	19
Age (M ± SD in years)	21.89 ± 2.42
Gender	9 females
Education (M ± SD in years)	16.21 ± 2.32
Handedness	19 right-handed

Note: M = mean; SD = standard deviation;

Consistency of resting-state FC, quantified by Sørensen-Dice similarity coefficient, is tabulated in Table 2 for the motor and language networks. While mostly comparable across many FC measures, the overlap coefficients for Pearson’s correlation are not necessarily the best in any of these tested networks. It was possible to validate the consistency for motor and language networks based on task functional MRI whereas the same could not be performed for other networks due to lack of corresponding task functional MRI in this cohort.

**Table 2:** Consistency of each measure of FC as measured by Sørensen-Dice similarity coefficient between task and resting-state conditions in three networks used in **E1**.

FC Measure	L-Motor vs Rest	R-Motor vs Rest	Language vs Rest
Correlation	0.502	0.466	0.508
Cross-correlation	**0.506**	0.496	0.492
Coherence	0.450	0.440	0.473
Wavelet coherence	0.500	**0.537**	0.525
Mutual information	0.3438	0.386	0.460
Euclidean distance	0.455	0.438	0.592
Cityblock distance	0.448	0.432	0.604
Dynamic time warping	0.444	0.446	0.584
Earth mover’s distance	0.497	0.514	**0.618**

**Note:** Functional networks defined based on Power atlas in **E1**. The highest overlap in each network is represented in bold.

An alternate procedure to evaluate the consistency of the resting-state FC at the whole-brain level was conducted by, first, simulating an *idealized* FC structure with a block structure along the diagonal as shown in Fig. 2 (subfigure B: c) and second, comparing the observed resting-state FC to it with Sørensen-Dice similarity coefficient between them. We refer to it as an idealized FC structure as it reflects maximum connectivity within any given network (block) and minimum connectivity between distinct networks (blocks) and allows us to compare the different FC measures. This is visually represented for Pearson’s correlation in Fig. 2 (subfigure B) (and for all other measures in Supplementary Fig. 2) and quantified for all FC measures in Table 3. Most of the FC measures showed comparable overlap between the idealized and observed FC pattern with Pearson’s correlation showing the highest overlap. This would then raise the following question: is it possible to do better than the current observed overlap? This is evaluated in the following experiments.

**Table 3:** Consistency of each measure of FC as measured by Sørensen-Dice similarity coefficient between the observed thresholded FC matrix (Fig. 2 (subfigure B: b)) and idealized thresholded FC matrix (Fig. 2 (subfigure B: c)) based on Power atlas in **E1**.

FC Measure	Observed vs Ideal FC
Correlation	**0.256**
Cross-correlation	0.252
Coherence	0.224
Wavelet coherence	0.239
Mutual information	0.180
Euclidean distance	0.225
Cityblock distance	0.225
Dynamic time warping	0.213
Earth mover’s distance	0.188

**Note:** The highest overlap is represented in bold.

### Experiment 2 (E2)

A typical application of FC has been in understanding the differences between population groups. The goal of this second experiment was to evaluate how well each FC measure performed in differentiating between younger and older healthy individuals across standard large-scale brain networks with a binary machine learning classifier. Additionally, the discriminatory power of a composite metric, obtained by combining all the FC measures, was tested.

### Findings from E2: Population-based classification using functional connectivity

The younger and older groups had a significant difference in age based on a two sample t-test but not in terms of gender, education, handedness and head motion as reported in Table 4. The goal, here, was to determine if this age-difference could be detected on the basis of resting-state FC of various brain networks. The support vector machine classifier models, implemented using neighborhood component analysis (NCA) feature selection and leave-one-out testing, were evaluated by the peak accuracy and area under the curve achieved. The accuracy levels are tabulated in Table 5 and area under the curve along with number of features used by each classifier are listed in the Supplementary Table 1. As seen in Table 5, Pearson’s correlation does not always stand out while differentiating the young from the old. Comparing performances of alternative FC, no one single FC measure particularly performed consistently better than the rest. Importantly, when we concatenate all these measures of FC, the composite measure almost always performs better or comparable to Pearson’s correlation. This could be due to the contribution of alternative FC measures which potentially augment the discriminatory power of Pearson’s correlation. The lack of consistency of the composite measure across all networks could be because the size of the training data could not be scaled up with increased number of features used for classification or that the definition of standard networks is not well-suited for the measure. While the former could not be tested due to availability of limited data, the latter was further tested in the final experiment.

**Table 4:** Characteristics of younger and older healthy participants included for **E2**.

Characteristic	Younger Healthy	Older Healthy	Group difference (*p*-value)
N	29	24	—
Age (M ± SD in years)	25.8 ± 7.880	58 ± 7.587	<0.001*
Gender	16 females, 13 males	16 females, 8 males	0.416
Education (M ± SD in years)	16.4 ± 2.244	16.9 ± 2.857	0.476
Handedness	29 right	24 right	1
Translation in x (M ± SD in mm)	0.038 ± 0.120	−0.022 ± 0.122	0.073
Translation in y (M ± SD in mm)	0.001 ± 0.330	−0.075 ± 0.290	0.373
Translation in z (M ± SD in mm)	0.003 ± 0.123	−0.009 ± 0.255	0.821
Rotation in x (M ± SD in degrees)	−0.014 ± 0.185	0.090 ± 0.304	0.128
Rotation in y (M ± SD in degrees)	−0.007 ± 0.089	-0.045 ± 0.153	0.270
Rotation in z (M ± SD in degrees)	−0.022 ± 0.067	−0.023 ± 0.071	0.951
Euclidean norm of motion	0.053 ± 0.021	0.067 ± 0.029	0.054
DVARS	26.224 ± 4.294	27.123 ± 5.974	0.527
FWD	3.001 ± 1.473	3.928 ± 1.929	0.052

**Note:** M = mean; SD = standard deviation; DVARS = spatial root mean square after temporal differencing; FWD = framewise displacement; *significantly different with *p*-value < 0.05.

**Table 5:** Age-based classification between younger and older healthy adults in nine major brain networks for **E2**.

FC Measure	D. DMN	V. DMN	L. ECN	R. ECN	A. Salience	P. Salience	Auditory	Language	Motor
Pearson’s Correlation	45.28%	56.60%	52.83%	**66.03%**	75.47%	62.26%	58.49%	58.49%	56.60%
Cross-correlation	54.72%	52.83%	54.72%	60.37%	79.24%	58.49%	58.49%	54.72%	54.71%
Coherence	54.71%	50.94%	50.94%	54.71%	60.37%	50.94%	58.49%	56.60%	**69.81%**
Wavelet coherence	49.06%	52.83%	52.83%	54.71%	67.92%	50.94%	62.26%	54.71%	52.83%
Mutual Information	62.26%	**75.47%**	62.26%	52.83%	77.35%	58.49%	52.83%	54.71%	54.71%
Euclidean distance	64.15%	69.81%	62.26%	**66.03%**	71.69%	50.94%	54.72%	54.71%	52.83%
Cityblock distance	**71.69%**	71.69%	64.15%	64.15%	83.01%	50.94%	52.83%	**64.15%**	54.72%
DTW	69.81%	73.58%	**69.81%**	62.26%	79.24%	56.60%	52.83%	60.37%	50.94%
EMD	52.83%	62.26%	64.15%	52.83%	66.03%	52.83%	52.83%	50.94%	66.03%
Composite	66.04%	69.82%	**69.81%**	54.72%	**84.91%**	**73.58%**	**69.81%**	58.49%	62.26%

**Note:** Brain networks are defined by the Willard functional atlas with a support vector machine classifier. Performance represents accuracy levels (%) with a leave-one out testing. The highest performing FC measure is represented in bold for each network. Additional performance measures are included in Supplementary Table 2; D.DMN = dorsal default mode network; V.DMN = ventral default mode network; L.ECN = left executive control network; R.ECN = right executive control network; A.Salience = anterior salience; P.Salience = posterior salience; DTW = dynamic time warping; EMD = earth mover’s distance.

### Experiment 3 (E3)

Classification between younger and older individuals in **E2** was performed on standard brain networks. However, the configuration of these standard brain networks are typically derived on the basis of Pearson’s correlation. In this final experiment, the aim was to test the potential dependence of the configuration of standard brain networks on FC measure. For this, a whole-brain (comprised of 10 standard networks) analysis was performed. Alternative brain configurations were obtained by applying unsupervised clustering for each FC measure. The biological plausibility of these alternative brain configurations was tested by investigating the association between FC and behavioral performance (verbal fluency) and compared with the standard brain configurations.

### Findings from E3: Large-scale brain configurations based on functional connectivity

The goal of this experiment was to inspect the assumption that resting-state FC may be organized in the form of a fixed number of networks, each involving a specific number of brain regions. While this may be the case for Pearson’s correlation, it may not necessarily be true for the alternative measures. Based on **E3**, the findings are:

(i) For every FC measure, the group mean whole-brain FC matrix representing the *original brain configuration* could be clustered into 10 major large-scale brain networks resulting in *alternative brain reconfigurations* for each FC measure. The *alternative brain reconfigurations* varied, with some better than the *original brain reconfiguration* and others worse based on the comparison of Sørensen-Dice similarity coefficient as can be observed in Supplementary Fig. 3.
(ii) A number of *alternative brain reconfigurations* demonstrated an improved Sørensen-Dice similarity coefficient (between each observed configuration and the corresponding expected idealized configuration) relative to the *original brain configuration* for each FC measure as reported in Table 6. The best possible *alternative brain reconfiguration* based on each FC measure demonstrated a greater dice overlap than the *original brain configuration*. Dynamic time warping distance had the greatest improvement (155.77%) while cross-correlation had the smallest improvement (13.15%). These two cases are visualized and compared in Supplementary Fig. 5. This pattern might suggest that the current predefined configuration may be optimal for correlation-based FC measure, however, the overall functional configuration of the brain may vary if the nature of the information captured by the FC measure deviates from correlation-based ones. Overall, reconfigurations based on mutual information, dynamic time warping distance and Earth mover’s distance did consistently better than the *original brain configuration* as seen from the distribution of the reconfigurations in Supplementary Fig. 3.

**Table 6:** Comparison of Sørensen-Dice similarity coefficient between the original configuration and the best possible reconfiguration of the brain via clustering (10 clusters) for E3.

FC Measure	Original Brain Configuration	Alternative Brain Reconfiguration
Pearson’s Correlation	0.460	0.528
Cross-correlation	**0.464**	0.525
Coherence	0.405	0.467
Wavelet Coherence	0.416	0.472
Mutual Information	0.212	0.337
Euclidean	0.316	0.361
Cityblock	0.311	0.441
Dynamic Time Warping	0.235	**0.601**
Earth Mover's Distance	0.259	0.433

**Note:** Cross-correlation and dynamic time warping exhibited the best overlap in the *original brain configuration* and best *alternative brain reconfiguration* respectively. A distribution of 1000 reconfigurations is visualized in Supplementary Fig. 4 by comparing the overlap with the original one. The FC measure showing the highest overlap in each case is indicated in bold text.
(iii) The plausibility of the best *alternative brain reconfiguration* was validated with the help of a data-inspired regression model in which the mean FC within each cluster/network for all participants was associated with the verbal fluency as a behavioral outcome. The goodness-of-fit (R^2^) was compared with that of the *original brain configuration* (Table 7) and can be found in Table 8. The best *alternative brain reconfiguration* associated with the verbal fluency scores was significant in some measures including Pearson’s correlation, wavelet coherence, and cross-correlation and marginally significant in some others such as Earth mover’s distance. This could imply, that subject to a larger sample size, there may exist an alternative functional rearrangement of the brain regions which may be indicative of behavioral outcomes.

**Table 7:** Brain-behavior relationship: Association (R^2^) between the mean FC within each of the 10 networks and the verbal fluency scores in 29 young healthy adults using the predefined original brain configuration as found with a stepwise regression model for **E3**.

Original Brain Configuration	D.DMN	V.DMN	L.ECN	R.ECN	A.Sal.	P.Sal.	Aud.	Lang.	Motor	Visual
Measure Showing Highest Association	Pearson’s correlation	Cross-correlation	Mutual information	EMD	Mutual information	Wavelet coherence	Cross-correlation	Cross-correlation	Pearson’s correlation	Cross-correlation
**R**^**2**^	**0.188**	**0.208**	0.027	0.047	0.015	0.107	0.098	0.022	**0.310**	0.106
***p*-value**	0.019*	0.013*	0.393	0.258	0.533	0.084^**†**^	0.099^**†**^	0.446	0.002*	0.085^**†**^

**Note:** Only the FC measure showing greatest associations with the outcome have been reported. Significant associations are represented in bold; D.DMN = dorsal default mode network; V.DMN = ventral default mode network; L.ECN = left executive control network; R.ECN = right executive control network; A.Sal. = anterior salience; P.Sal. = posterior salience; Aud. = auditory; Lang. = language; DTW = dynamic time warping; EMD = earth mover’s distance; R^2^: coefficient of determination; *association is significant with *p*-value < 0.05; ^**†**^association is marginally significant with *p*-value < 0.1.

**Table 8:** Brain-behavior relationship: Association (R^2^) between each cluster (C1 through C9) of the best **alternative brain reconfiguration** found by clustering and the verbal fluency score for each FC measure with a stepwise regression model for **E3**.

FC Measure	C 1	C 2	C 3	C 4	C 5	C 6	C 7	C 8	C 9	C 10
Pearson’s Correlation	0.013	0.031	0.197*	0.042	0.051	0.001	0.036	0.233*	0.091	**0.109**^**†**^
Cross-correlation	0.024	0.027	0.072	0.073	**0.103**^**†**^	0.081	0.043	0.011	**0.106**^**†**^	0.208*
Coherence	0.001	0.002	0.002	0.001	0.000	0.000	0.025	0.001	0.074	0.002
Wavelet coherence	0.069	0.000	0.021	0.009	0.060	0.026	0.145*	0.017	0.006	0.022
Mutual information	0.023	0.020	0.001	0.016	0.019	0.014	0.015	0.051	0.018	0.006
Euclidean	0.023	0.068	0.009	0.059	0.019	0.023	0.072	0.011	0.012	0.055
Cityblock	0.031	0.014	0.013	0.027	0.007	0.022	0.031	0.001	0.024	0.055
DTW	0.012	0.009	0.005	0.005	0.007	0.031	0.023	0.014	0.032	0.012
EMD	0.002	0.006	0.022	0.004	0.015	0.000	0.004	0.001	**0.110**^**†**^	0.045

**Note:** C1 through C9 represent clusters obtained by k-means; DTW = dynamic time warping; EMD = Earth mover’s distance; *represent association is significant with *p*-value < 0.05; ^**†**^represent association is marginally significant with *p*-value < 0.1.
(iv) Parallel to the multi-metric approach described in **E2** for the purpose of population-based classification, the same approach was extended for the prediction of behavioral outcomes. The results are tabulated in Table 9. Similar to results of classification, the combined FC measure was more consistent than individual measures in correlating with the behavioral outcome. The composite multi-metric representation of FC from the *alternative brain reconfiguration* performed better than that from the *original brain configuration* which may suggest that there is room for a better design of the functional configuration of the brain on the basis of measures adopted to quantify FC relative to the conventional correlation-based large-scale brain configuration.

**Table 9:** Brain-behavior relationship: Comparison between brain-behavior relationship of the **original brain configuration** and the best **alternative brain reconfiguration** using the composite multi-metric definition of FC as found with a stepwise regression model for **E3**.

	*Original Brain Configuration* Composite FC ~ Verbal Fluency	*Alternative Brain Reconfiguration* Composite FC ~ Verbal Fluency
R^2^	**0.495**	**0.606**
*p*-value	0.026*	0.0014*
Measures Selected	Coherence: Wavelet Coherence*	Dynamic Time Warping*
	Mutual Information*	Euclidean*
	Dynamic Time Warping*	Pearson’s Correlation: Earth mover’s distance
	Euclidean*	Cityblock
	Cross-correlation	Pearson’s Correlation
	Wavelet Coherence	Earth mover’s distance
	Coherence	

**Note:** R^2^ = coefficient of determination;:interaction term; *associated *p*-value < 0.05.
(v) A comparison of brain regions involved in the composite multi-metric FC in the *original brain configuration* and the *alternative brain reconfiguration* are presented in Table 10. Specifically, in the *original brain configuration*, the specific network, whose FC demonstrated the strongest association with the verbal fluency behavioral outcome varied by measures used by the stepwise regression model. A dominance of DMN (dorsal and ventral) was observed which is presumably the most robust network in resting-state functional MRI. In the *alternative brain reconfiguration*, however, the cluster whose FC demonstrated the highest association with verbal fluency was comprised of brain regions from various standard networks. As an example, for dynamic time warping (used by the stepwise regression model), the cluster that showed peak association with verbal fluency consisted of two regions of the standard language network and one region of the dorsal DMN. This could imply that the arrangement of brain regions that are most indicative of behavior may be variable by FC measure in question.

**Table 10:** Brain-behavior relationship: Distribution of brain regions involved in the networks/clusters of the **original brain configuration** and the best **alternative brain reconfiguration** for **E3**.

*Original Brain Configuration*: Composite FC ~ Verbal Fluency		*Alternative Brain Reconfiguration*: Composite FC ~ Verbal Fluency	
FC Measures Involved	Brain Regions Involved	FC Measures Involved	Brain Regions Involved
Coherence: Wavelet Coherence*	D.DMN: V.DMN	Dynamic Time Warping*	Language_2_ + D.DMN_1_
Mutual Information*	V.DMN	Euclidean*	Auditory_1_ + Language_1_ + D.DMN_2_ + P.Sal_3_
Dynamic Time Warping*	Motor	Pearson’s Correlation: Earth mover’s distance	Language_3_: Motor_3_ + RECN_1_ + V.DMN_3_
Euclidean*	D.DMN	Cityblock	LECN_1_ + Language_1_ + Motor_1_ + A.Sal_1_ + P.Sal_1_ + D.DMN_1 + _V.DMN_1_
Cross-correlation	Auditory	Pearson’s Correlation	Language_3_
Wavelet Coherence	V.DMN	Earth mover’s distance	Motor_3_ + RECN_1_ + V.DMN_3_
Coherence	D.DMN		

**Note:** interaction term; *indicate that the associated *p*-value < 0.05; D.DMN = dorsal default mode network; V.DMN = ventral default mode network; L.ECN = left executive control network; R.ECN = right executive control network; A.Sal. = anterior salience; P.Sal. = posterior salience; subscripts in the last column indicate the number of regions drawn from a standard brain network in the cluster whose mean FC was best associated with verbal fluency outcome.

## Discussion

In light of preliminary evidence based on the resting-state functional MRI data used in this study, two major hypotheses were formulated:

### Hypothesis 1: Functional connectivity could be better characterized with a multi-metric representation

Using FC derived from resting-state functional MRI, it was possible to not only perform population-based classification in **E2** but also regression to study the relationship between FC and behavioral outcome in **E3**. A comparison of contribution of all FC measures in these experiments showed that there was not necessarily one single FC measure that consistently outperformed others. However, combination of multiple measures by concatenation followed by a feature selection procedure was relatively more consistent and, in most cases, performed better than Pearson’s correlation. Thus, the first hypothesis suggests that a more complete measure of FC could be developed by combining information from multiple measures. This would be advantageous as it would augment the correlation-based FC with complementary measures which capture linear, non-linear, similarity, dissimilarity, time-, frequency- and wavelet-domain properties and interactions between the signals.

### Hypothesis 2: Canonical brain network configurations are metric-dependent

Decomposition of the whole brain into component networks is a way to understand interacting regions functioning in synchronization which may be responsible for specific traits and/or behavior. However, these networks/clusters are most conventionally based on a correlation-based measure. **E3** suggested that the same set of regions in the brain may be rearranged and clustered into alternative brain configurations with networks/clusters distinct from those defined for Pearson’s correlation. The variation in *alternative brain reconfigurations* by FC measure may signal that the large-scale brain networks are a function of the measure that quantifies synchronization among the regions. Since the alternative measures explored in this study elicit information complementary to that by Pearson’s correlation, it is likely that the functional connectomic view of the brain would be variable.

### The big picture

Ideas of segregation and integration in the brain are well established^17,18^. It is, however, important to acknowledge that there are individual variations of a specific integrated component which may be lost during group-level analyses. Additionally, connectivity typically associated with a particular entity is not necessarily always unique. For instance, the sites of the brain included within the default mode network are not always connected with the same strength across individuals or even within individuals^19^, which was confirmed in this work by means of a contrary case presented in Fig. 1.

A growing number of prior studies have indicated the need for characterizing FC using alternative measures. The advantages of harnessing both temporal and spectral information has been illustrated with the use of wavelet coherence to capture non-stationarity in BOLD signals in resting-state functional MRI^20,21^ and for population-based classification^22^. Mutual information, which could be interpreted as the amount of information flowing between the given regions, has been shown to perform better in the context of task functional MRI^23^ as well as resting-state functional MRI^24,25^. Dynamic time warping has been demonstrated to capture the non-stationarity in simulated functional MRI data^26,27^. The importance of non-linear and directional dependencies among BOLD signals is highlighted by means of mutual connectivity^28^.

The present study adds to these works by comparing and contrasting multiple alternative FC measures and investigating not only the neural interactions differing between subgroups in a given population but also brain-behavior relationships arising from these measures. The goal of this work is to encourage development of a more holistic view of functional connectivity rather than reliance on a single measure.

### Methodological considerations

While this study outlines a number of ways of quantifying FC, it is important to recognize the assumptions and choices made in the experiments which may have bearing on the current findings. First, the number of samples used to investigate effects varied from 19 in **E1**, to 53 in **E2**, to 29 in **E3**. Evaluation of research questions in these relatively different but overlapping datasets offers confidence in the findings to some degree. However, it is important to acknowledge that these are modest sample sizes. This study should be considered as a proof-of-concept and the generalizability of the effects found here would need to be substantiated in much larger samples in healthy as well as pathological population groups.

Second, in weighing out the contribution of the various FC measures in **E2** and **E3**, 10 major large-scale networks were considered. These tests assume a priori that the FC in the whole brain can largely be divided into 10 groupings. While this may be the optimal number for Pearson’s correlation, it may not be appropriate for the alternative FC measures. In **E3**, we attempted to decompose the brain into 10 distinct brain networks via clustering based on the a priori knowledge that the whole brain FC matrix (e.g. in Supplementary Fig. 4) is comprised of roughly 10 major networks. However, results specified in Table 6, when visualized (Supplementary Fig. 5), point toward the possibility that the number of decomposable networks might also vary by FC measure, in addition to the structure of each network. In Supplementary Fig. 5 (subfigure a: i), the FC matrix of the *original brain configuration* for cross-correlation (which showed the best overlap across all measures) shows a block-like diagonal structure corresponding to 10 networks. However, in Supplementary Fig. 5 (subfigure b: iii) the FC matrix of the *alternative brain reconfiguration* for dynamic time warping (which showed the best overlap across all measures) shows a block-like diagonal structure corresponding to about 4 potential networks. A possible approach to examining this could involve application of spatio-temporal independent component analysis^21^ to each measure. Additionally, it must be noted that in deriving the *alternative brain reconfigurations*, the size of the clusters determined by k-means clustering was not controlled for. Considering the likelihood that changing the definition of FC the brain connectome could lead to variation in not only the pattern of brain connectome, but also the number and size of decomposable brain networks, further examination is warranted.

Thirdly, included within this investigation were FC measures which capture undirected interactions between BOLD signals under the supposition that the dependency between BOLD signals arising from two brain regions is symmetric. However, BOLD signals need not necessarily adhere to this. Multi-metric approach presented here could be supplemented and informed better by incorporating directional influences capturing causality such as effective connectivity^17^, interactions between two regions in presence of signals arising from other regions with measures such as partial correlation^29^ or generalized coherence^30^ and reducing the effects of unobserved or latent sources such as differential covariance^31^.

Fourth, the premise behind organized networks in the brain was based upon achieving an idealized brain configuration with a tight block-like structure along the diagonal of a symmetric FC matrix (such as the one in Supplementary Fig. 4) which represents a structure that is strongly connected within a given network (shown in beige color; value = 1) and weakly connected between networks (shown in black color; value = 0). Whether this is a desirable idealized brain configuration needs further investigation. In other words, a question to explore would be: should distinct brain networks be treated as independent groupings of brain areas operating in synchrony or should there be a certain level of dependence between networks?

Finally, to enable a fair comparison across FC measures, only the absolute magnitude of each was utilized. Some measures, however, have additional properties that may be useful in understanding BOLD interactions better. For instance, coherence offers the specific frequency and wavelet coherence offers both temporal and spectral instances at which maximum similarity is observed. These additional details could enhance the characterization of FC and should be considered in subsequent studies.

### Future directions

As a pilot study pointing towards a comprehensive multi-metric notion of FC, this study holds promise for further exploration in several directions, some of which are outlined as follows:

(i) Only a small number of alternative FC measures were studied here. Although these covered a wide range of properties by encompassing measures of similarity, dissimilarity, from time-, frequency- and wavelet-domains, captured linear and non-linear relationships among BOLD signals, there may be other measures to better capture FC.
(ii) The idea of a composite definition was realized and executed by a straightforward concatenation of the distinct FC measures. Forming a multi-metric representation could be approached by alternate means such as identification of a linear, quadratic or higher order, log transformation, weighting, or convolutional method, of combining the FC measures^19^.
(iii) A majority of the studies in the neuroimaging literature have relied upon an elementary design of FC by considering pairwise BOLD interactions of nodes. Future studies should move from pairwise FC towards generalized FC to gain a clearer picture of the brain connectome by considering multi-nodal models and analyzing BOLD interactions among a group of nodes (more than a pair) simultaneously (similar to interpretations given by network-based statistic toolbox^12^).
(iv) The introduction of multiple measures requires a deeper understanding of their properties, especially if they are likely to capture complementary information of the same signal. This could entail studying of statistical properties, dependence on noise in the signal, and the sensitivity to outliers of each FC measure.

## Methods

### Conventional characterization of functional connectivity

One approach to characterize integration is in terms of FC, which is usually inferred on the basis of correlations among measurements of neuronal activity of anatomically separated regions. FC, originally defined as the statistical dependencies among remote neurophysiological events in positron emission imaging^4^, was subsequently applied to functional MRI data^5^. In most applications, the convention has been to use Pearson’s correlation as it is simple to quantify and has an intuitive interpretation. However, statistical dependence between signals can arise in a variety of ways.

This has been depicted in Fig. 1 within the context of FC. Essentially, a fail/contrary case is presented by considering two functionally connected brain regions, assumed to be part of the standard DMN in the healthy brain. At a single-subject level, examination of the neurophysiological events (BOLD signals) associated with these regions appear to have a low level of correlation as captured by the time-domain similarity of Pearson’s correlation, on a scale of 0 to 1. However, similarity between signals arising from the same regions in the frequency-domain (quantified by magnitude-squared coherence) and wavelet-domain (quantified by wavelet coherence) are comparatively higher (these are also presented on the scale of 0 to 1). This case illustrates that a low correlation in time-domain must not be mistaken for no correlation and can also be supported mathematically. Rather, it should be treated as lack of linear time-dependence only. This could imply that there may still be dependencies between these BOLD signals, that are not captured well by Pearson’s correlation. Capturing the true underlying dependencies is an essential task in the full understanding of brain connectivity.

### Are there alternative measures to quantify functional connectivity?

Based on a literature review within the neuroimaging and signal processing disciplines, a number of measures were identified that capture statistical dependence between two signals. For all subsequent experiments, suppose the following. Based on the preprocessed functional MRI data for any subject, BOLD time-series signal can be extracted from any region in the brain which lasts for *t*− timepoints. Assuming that a network of interest in the brain is comprised of *n*− regions, we would have a *t* × *n* matrix, where each column vector would represent the BOLD time-series for a given region, each with *t*− timepoints. Variables *x* and *y* would represent time series from any pairs of distinct regions with each $x,y\in {{\mathbb{R}}}^{t}$. Pairwise FC, measuring the statistical dependence between all possible pairs in a given network would yield a matrix of size *n* × *n*. This would generate a symmetric matrix and can be reduced to $\frac{n(n-1)}{2}$ unique coefficients (from either the upper or lower triangle of the matrix). The following defines and characterizes each identified measure quantifying FC, a summary of which is presented in Supplementary Table 2.

#### Pearson’s correlation

Pearson’s Correlation is a similarity measure and provides a relative measure of association between two signals^32^ and is given by:

1 $${\rho }_{corr}(x,y)=\frac{cov(x,y)}{\sqrt{var(x)var(y)}}=\frac{(x-\overline{x}){(y-\overline{y})}^{T}}{(\sqrt{(x-\overline{x}){(x-\overline{x})}^{T}})\{\sqrt{((y-\overline{y}){(y-\overline{y})}^{T})}\}}$$

where *cov*(*x*, *y*) is the covariance between signals, $\overline{x}$ and $\overline{y}$ are the mean values of the respective signals, *var* (*x*) *and var* (*y*) represent the variance of each signal respectively.

The quantity ρ_*corr*_ captures the linear relationship between the signals, is bounded above by 1 in absolute value and is scale-invariant in magnitude. A positive value indicates that the time series signals tend to be simultaneously greater than their respective means. And a negative value implies that the signals tend to fall on opposite sides of their respective means. The absolute value reflects the strength of the tendency to be above or below their means. A value closer to 0 suggests that the signals are uncorrelated in terms of a linear correlation. This implies that a linear relationship is not enough to capture the true relationship between them; it should not be treated as evidence for an absence of a relationship.

#### Cross-correlation

Cross-correlation^33^, a similarity measure, could simply be considered as the extended version of Pearson’s correlation as it calculates the linear correlation between all possible shifted versions of a signal relative to the other signal as follows:

2 $${\rho }_{cross-corr}(x,y)={\rho }_{xy}(m)=\left\{\begin{array}{cc}{\sum }_{i=0}^{t-m-1}{x}_{i+m}{y}_{i}^{\ast } & if\,m\,\ge \,0\\ {\rho }_{yx}(\,-\,m) & if\,m\, < \,0\end{array}\right.$$

where ${y}_{i}^{\ast }$ represents the complex conjugate of *y*_*i*_. Index *m* is the displacement between the two signals and is called a lag or lead depending on whether it assumes a positive or negative value.

Since it computes the correlation between displaced versions of two signals, *ρ*_*cross-corr*_ ranges from -1 to 1 and must be interpreted just like linear correlation. While correlation between two signals generates a single similarity measure, cross-correlation generates a vector of similarity measures corresponding to each value of *m*. The maximum value of this vector for a particular *m* can be used as a feature for further analysis. This could be useful in identifying regions of the brain that might not be functionally connected at the same time but be functionally connected after a lag period.

#### Coherence

The spectral coherence^34^ allows assessment of the correlation or similarity between two signals in the frequency-domain. Also known as the magnitude-squared coherence, its value indicates how similar *x* and *y* are at each frequency. Coherence can be expressed as:

3 $${\rho }_{coherence}(x,y)=\frac{| {P}_{xy}(f){| }^{2}}{{P}_{xx}(f){P}_{yy}(f)}$$

where *P*_*xx*_ (*f*) and *P*_*yy*_(*f*) are the power spectral densities of *x* and *y* respectively and *P*_*xy*_ (*f*) is the cross-power spectral density of *x* and *y*.

The value of coherence lies between 0 and 1, with 0 indicating no coherence between the signals and 1 indicating strong coherence between the signals. It can be considered to reflect the phase consistency between two signals at a given frequency. On one hand, a weaker coherence is the case when the signals share a random phase relationship and on the other hand, stronger coherence results when the phase relationship is almost constant between the signals. Since a coherence value is obtained for each frequency component present in the signals, the peak similarity achieved could be utilized for further analysis.

#### Wavelet coherence

Wavelet coherence^35^ captures similarity and quantifies how time signals from two sources are related in the time-frequency-domain. It is based on computing the cross-wavelet power which reveals the parts of the signals that share high common power. Wavelet coherence measures the coherence of the cross wavelet transform in time-frequency-domain and is given by:

4 $${\rho }_{wcoherence}(x,y)=\frac{{|S({C}_{x}(a,b){C}_{y}(a,b))|}^{2}}{S({|{C}_{x}(a,b)|}^{2})S({|{C}_{x}(a,b)|}^{2})}$$

where *S* is the smoothing operator in time and scale, *C*_*x*_(*a*, *b*) *and* (*C*_*y*_ (*a*,*b*)) represent the continuous wavelet transform of *x* and *y* at scales *a* and positions *b* respectively. For real-valued signals, *ρ*_*wcoherence*_ (*x*, *y*) would be real-valued by choosing real-valued wavelets. Comparing the forms presented in Eq. (4) and Eq. (1) suggests that *ρ*_*wcoherence*_ could be considered as the equivalent of *ρ*_*corr*_ in the time-frequency domain. The magnitude of *ρ*_*wcoherence*_ can vary between 0 (no similarity) and 1 (identically similar). Unlike in *ρ*_*cross-corr*_ or *ρ*_*coherence*_, *ρ*_*wcoherence*_, requires computation of a similarity measure at each point on the two-dimensional time-frequency plane. Subsequent analysis could be carried out by choosing the greatest similarity value corresponding to a particular time and frequency.

#### Mutual information

Inspired by information theory, if *x* and *y* were to be treated as discrete random variables over the space ${\mathscr{X}}\times {\mathscr{Y}}$, then the similarity in the form of mutual information^36^ between them can be defined as:

5 $${\rho }_{mutual{\rm{\_}}info}(x,y)=I(x;y)=\sum _{y\in {\mathscr{Y}}}\sum _{x\in {\mathscr{X}}}p(x,y)log\frac{p(x,y)}{p(x)p(y)}$$

where *p*(*x*,*y*) is the joint probability mass function of *x* and *y*, *p*(*x*) *and p*(*y*) are the marginal probability mass functions of *x* and *y* respectively.

Essentially, *ρ*_*mutual_info*_ captures the information that is shared between *x* and *y*, i.e., it measures how much knowing one of them reduces uncertainty about the other. *ρ*_*mutual_info*_ can assume non-negative values only. On one hand, if *ρ*_*mutual_info*_ = 0, then knowledge of *x* does not offer any knowledge of *y* and vice-versa. On the other hand, if there exists a deterministic relationship between *x* and *y*, then knowledge of *x* is also shared with *y* and vice-versa. In this case, *ρ*_*mutual_info*_ is equivalent to the entropy of each *x* as well as *y* which represents the expected information stored by each random variable.

#### Euclidean distance

Euclidean distance is a dissimilarity measure and one of the more commonly used metrics due to the well-studied background of Euclidean spaces. It is easy to conceptualize and intuitive as it measures the geometric distance between two points. In case of vectors, this can be computed by the following:

6 $${\rho }_{euclidean}(x,y)={\Vert x-y\Vert }_{2}=\sqrt{(x-y){(x-y)}^{T}}$$

The difference terms serve as the measure of similarity. *ρ*_*euclidean*_ is dependent on the magnitude of individual points of the vectors. While it is bounded below by 0 indicating low dissimilarity, there is no upper bound. However, it can be rescaled to range between 0 and 1 for interpretability. Euclidean distance is not invariant to the scale of the data. It must be applied once the data has been appropriately scaled.

#### Cityblock distance

Cityblock distance is derived by looking at the difference in absolute values in each dimension of the signals and represents dissimilarity between them. It is given by:

7 $${\rho }_{cityblock}(x,y)=\Vert {x-y\Vert }_{1}={\sum }_{{\rm{i}}=1}^{t}|{{\rm{x}}}_{{\rm{i}}}-{{\rm{y}}}_{{\rm{i}}}|$$

*ρ*_*cityblock*_ can decompose the contributions made by each variable of the signal in terms of the difference in their absolute values. As with *ρ*_*euclidean*_, the measure *ρ*_*cityblock*_ is bounded below by 0, is not bounded above and scale-variant. Values closer to 0 are more desirable to claim lower dissimilarity between vectors. Unlike *ρ*_*euclidean*_, which squares the difference in amplitudes and amplifies the deviation, the larger differences in *ρ*_*cityblock*_ are not amplified.

#### Dynamic time warping

Dynamic time warping^37^ measures dissimilarity and provides an alignment between the signals by means of non-linear warping of the time axis. This metric is based on evaluating a local cost of similarity between all possible pairs of dimensions between two signals and creating a lattice. Based on this lattice, the signals are aligned so as to have the maximum overall overlap (or minimum cost in the optimization framework). The steps involved are as follows:

For two signals, *x* and *y*, let *ρ*_*ij*_ be the Euclidean distance between *i*^*th*^-dimension of *x* and *j*^*th*^-dimension of *y*. All pairwise distances *ρ*_*ij*_ are arranged into a lattice C_i,j_(*x*, *y*) of size *t* × *t*. Then *ρ*_*dtw*_ searches through the lattice for a path parameterized by two sequences of the same length such that

8 $${\sum }^{}{{\rm{C}}}_{{\rm{i}},{\rm{j}}}(x,y)$$

is minimum. The chosen path is such that both signals are aligned, without skipping dimensions and without repetition of signal dimensions. Any non-linear variation in the time-domain are taken into account here. Being a dissimilarity measure, dynamic time warping is bounded below by 0 and unbounded above. It is also scale-variant and is applicable to the general case where signals are of varying lengths in time although in case of BOLD signals, the length of the signals are the same.

#### Earth mover’s distance

Earth mover’s distance, also known as Wasserstein metric^38^, is a dissimilarity measure which assumes each signal to be a probability distribution and represents the minimum cost of converting one distribution into the other. Treating *x* and *y* as probability distributions with:

$$x=\{({t}_{{x}_{1}},{x}_{1}),({t}_{{x}_{2}},{x}_{2}),\ldots ({t}_{{x}_{m}},{x}_{m})\}$$

and

9 $$y=\{({t}_{{y}_{1}},{y}_{1}),({t}_{{y}_{2}},{y}_{2}),\ldots ({t}_{{y}_{n}},{y}_{n})\}$$

where each *x*_*i*_ is a cluster (=amplitude) of the signal *x* at time-point ${t}_{{x}_{i}}$ and each *y*_*j*_ is a cluster (=amplitude) of signal *y* at time-point ${t}_{{y}_{j}}$. While *m* and *n* do not have to be necessarily equal in general, i.e., Earth mover’s distance can be computed for signals of differing lengths, in case of BOLD signals, they can be considered to be the same for a given individual and determined by the scan length. Then the ground distance between clusters at *p*_*i*_ *and q*_*j*_ can be encoded in the matrix

10 $$D=[{d}_{i,j}]$$

with a flow between clusters at *p*_*i*_ *and q*_*j*_ represented by the matrix

11 $$F=[{f}_{i,j}]$$

The objective is to minimize the overall cost

12 $${\min }\,{\sum }_{i=1}^{m}{\sum }_{j=1}^{n}{f}_{i,j}{d}_{i,j}$$

while satisfying the following constraints:

13 $${f}_{i,j}\ge 0\,for\,1\le i\le m,\,1\le j\le n$$

14 $${\sum }_{j=1}^{n}{f}_{i,j}\le {t}_{{x}_{i}}\,for\,1\le i\le m$$

15 $${\sum }_{i=1}^{m}{f}_{i,j}\le {t}_{{y}_{j}}\,for\,1\le j\le n$$

16 $${\sum }_{i=1}^{m}{\sum }_{j=1}^{n}{f}_{i,j}=\,{\min }\left\{{\sum }_{i=1}^{m}{t}_{{x}_{i}},{\sum }_{j=1}^{n}{t}_{{y}_{j}}\right\}.$$

Earth mover’s distance can then be defined as the amount of work needed to transform distribution *x* to distribution *y*, normalized by the total flow

17 $${\rho }_{emd}(x,y)=\frac{{\sum }_{i=1}^{m}{\sum }_{j=1}^{n}{f}_{i,j}{d}_{i,j}}{{\sum }_{i=1}^{m}{\sum }_{j=1}^{n}{f}_{i,j}}$$

Similar to *ρ*_*dtw*_, *ρ*_*emd*_ considers non-linear interactions between signals, is scale-variant, and applicable to general signals of unequal length. This measure is scale-bounded below by the distance between the centroids of the distributions or signals and values closest to it represent greater similarity.

#### Implementational details

These measures were computed in a pairwise fashion, i.e. a FC value was obtained for each pair of regions included in each experiment. The specifications of implementation (in MATLAB R2018a) are provided in Supplementary Table 2.

### Experiments

Three experiments were designed to closely assess each of the alternative measures relative to Pearson’s correlation as well as to compare them to each other:

#### Experiment 1 (E1)

The consistency of each FC measure was evaluated by comparing task and resting-state functional MRI in specific brain networks.

#### Experiment 2 (E2)

The contribution of each FC measure was studied in an age-based classification problem in several standard large-scale brain networks.

#### Experiment 3 (E3)

The potential dependence of brain organization into large-scale networks was tested for each FC measure.

For all the experiments in this section, imaging data (i.e., task and resting-state functional MRI) were acquired and preprocessed similarly. The commonalities across the experiments are described as follows:

### Participants

All healthy adults who participated in this study provided written and informed consent. The data collection protocol was approved by the University of Wisconsin-Madison Health Science Institutional Review Board and the study was carried out in accordance with the relevant guidelines and regulations.

### Data acquisition

Neuroimaging data were acquired from recruited participants on 3T GE 750 scanners (GE Healthcare, Waukesha, WI, USA) with an 8-channel head coil. An axial localizer scan was obtained to verify subject positioning and plan slice acquisition.

#### Structural MRI

Five-minute T1-weighted axial structural images were acquired at the beginning of each session using FSPGR BRAVO sequence (TR = 8.132 ms, TE = 3.18 ms, TI = 450 ms, 256 × 256 matrix, 156 slices, flip angle = 12°, FOV = 25.6 cm, slice thickness = 1 mm).

#### Task functional MRI

Each task functional MRI followed a block design consisting of four 20-s task blocks alternating with five 20-s blocks of rest, for a total scan time of 3 minutes. The first followed a finger tapping task paradigm targeted at capturing motor network activation in which participants alternated between tapping fingers on a button box sequentially and continuously and resting, based on visual cues. The second used a verbal fluency task functional MRI paradigm aimed at capturing language network activation in which participants alternated between covertly verbalizing words starting with a given letter (“F”, “A”, “S”, “T”) and resting based on visual cues. Participants used earplugs to attenuate scanner noise, were padded with foam pads around their head and were instructed to hold their heads still during the scan in order to minimize movement.

#### Resting-state functional MRI

Ten-minute resting-state functional MRI were obtained using single-shot echo-planar T2*-weighted imaging with the following acquisition parameters: TR = 2.6 s, 231 time-points, TE = 22 ms, FOV = 22.4 cm, flip angle = 60°, voxel dimensions 3.5 mm × 3.5 mm, 3.5 mm slice thickness, 40 slices, with eyes closed. Two types of task functional MRI were collected via echo-planar T2*-weighted imaging either with the same parameters as the resting-state scan or with parameters: TR = 2.0 s, 90 time-points, TE = 22 ms, FOV = 22.4 cm, flip angle = 60°, voxel dimensions 3.75 mm × 3.75 mm, 4.0 mm slice thickness, 40 slices.

### Data preprocessing

All imaging data were preprocessed on AFNI^39^ using standard steps as described below.

#### Task functional MRI

For each of the task functional MRI (left motor, right motor, language), data were first aligned to the anatomical and normalized to standard Montreal Neurological Institute (MNI) space. The first four volumes were discarded to allow for steady-state imaging. Images were then resampled to 3.0 mm isotropic, de-spiked, volume registered, and spatially smoothed using a 4 mm full-width at half-maximum Gaussian kernel. Time course at each voxel was scaled to percent signal with a mean value of 100. The standard activation maps were computed using a general linear model (GLM) with a canonical gamma variate hemodynamic response function convolved with a boxcar reference waveform and six rigid-body motion parameters and their derivatives regressed. Motion censoring (per TR motion > 0.25 mm) was included in the general linear model. Standard activation maps were also derived using AFNI’s 3dClustSim (*p* < 0.05, ≥20 voxels).

#### Resting-state functional MRI

Data were de-spiked, slice time corrected, motion corrected, aligned with the structural MRI, normalized to MNI space, resampled to 3.5 mm^3^, and spatially smoothed with a 4-mm FWHM Gaussian kernel. Motion censoring (per TR motion > 1 mm or 1°), nuisance regression, and bandpass filtering (0.01–0.1 Hz) were performed simultaneously in one regression model. Nuisance signals regressed out included six motion estimates and their temporal derivatives, and the voxel-wise locally averaged white matter signal. Global signal regression was not performed.

### Data analysis

All data analyses to follow were carried out with the Statistics and Machine Learning Toolbox in MATLAB R2018a (The MathWorks, Inc., Natick, Massachusetts, United States).

### E1: Consistency of functional connectivity

#### Objective

In order to understand whether the alternative measures capture FC based on resting-state data, it is important establish the ground truth FC for each. Given that the spatial FC patterns derived from resting-state BOLD signals have been reported to overlap with those derived from task-based data across brain networks and individuals, task-functional MRI was used to represent ground truth^5,40–42^. Thus, the FC derived from resting-state data was compared with the FC derived from task-functional MRI data to evaluate the consistency of each measure.

#### Participants

Neuroimaging data were acquired from 22 young healthy right-handed participants (age = 18–28 years). Of the 22 participants, 3 were excluded due to excessive head movement as a result of motion censoring leading to too few degrees of freedom, leaving data from 19 participants for subsequent analyses. Detailed demographic information for included participants can be found in Table 1.

#### Data analysis

BOLD time courses were extracted from preprocessed task functional MRI and resting-state functional MRI data in the motor and language networks based on standard functional (Power) atlas^43^. For the left and right finger-tapping task functional MRI, the motor network, comprised of 35 regions, was used. For the verbal fluency task functional MRI, the working memory and fronto-parietal sub-networks were combined to include 30 regions. All these networks were also evaluated for resting-state functional MRI. Within each network, pairwise FC was evaluated, generating a 35 × 35 and 30 × 30 matrix for motor and language (working memory and fronto-parietal areas) functions respectively for each individual.

FC matrices for all individuals were averaged to generate a group-level mean FC matrix for each network. This meant that each element in the average FC matrix was obtained by computing the mean of FC values in the cell of each of the 19 individual matrices. Then the mean group-level FC matrix was thresholded to reveal a binarized FC pattern within a given network. The threshold was empirically determined to be one standard deviation higher than the mean overall (all values pooled across subjects) FC value so as to have stronger within-network and weaker between-network connectivity. Binarized FC patterns were compared between task functional MRI and resting-state functional MRI by computing a Sørensen-Dice similarity coefficient^44,45^ which measures the consistency of each of the alternative FC measures.

### E2: Population-based classification using functional connectivity

#### Objective

After examining the consistency of the various measures of FC, a data-driven comparative analysis was performed to further evaluate the alternative measures. This consisted of population-based, specifically age-based, classification in the healthy population. The goal was to compare and contrast the different FC measures at the brain network-level in differentiating between younger brains from older ones.

#### Participants

Neuroimaging data were acquired from 64 healthy right-handed participants, subdivided into 32 older (age = 46–74 years) and 32 younger (age = 18–45 years) participants. The two groups differed significantly by age but were matched in terms of gender distribution, education, verbal fluency and head motion to avoid these possible confounds. These criteria led to the exclusion of 8 older and 3 younger adults, leaving 24 older and 29 younger participants for further analysis (*n* = 53). Greater head movement in older adults may be a potential reason for exclusion of a greater number^46^. Group-wise characteristics are provided in Table 4.

#### Data analysis

BOLD time courses were extracted from preprocessed resting-state functional MRI in 9 major brain networks based on a second standard functional (Willard) atlas^8^ as it has a specific language network defined (used in subsequent analysis). The networks included dorsal and ventral default mode networks (D. DMN, V. DMN), left and right executive control networks (L. ECN and R. ECN), anterior and posterior salience networks (A. Salience and P. Salience), auditory, language and motor networks. Within each network, pairwise FC was evaluated, generating a symmetric square matrix for each individual.

Each network-based FC matrix was vectorized to extract only the unique pairwise FC coefficients ($\frac{n(n-1)}{2}$, where *n* = number of regions of interest in the network) for each participant (from either the upper or lower triangular matrix). Then these vectors were compiled across subjects (feature size = $53\times \frac{n(n-1)}{2}$) and fed into a binary support vector machine classifier. A nested cross-validation approach, comprised of two loops, with leave-one-out strategy was adopted to maximize the training dataset. The inner loop determined an optimal number of discriminatory FC features with NCA (neighborhood component analysis) which is a non-parametric and embedded feature selection approach^47^. It selects features (a subset of $\frac{n(n-1)}{2}$ features) by learning a linear transformation which maximize the leave-one-out classification performance. The outer loop was used to perform model selection by classifying an independent left-out subject with the NCA-chosen features. The classification label (i.e. group membership) across all the left-out subjects was considered to compute the average accuracy and the classification probabilities of the left-out-samples were used to compute the area under the curve. These steps were repeated for each of the FC measures and results were compared. In addition to examining the discriminatory power of each FC measure separately, the above steps were also repeated by combining (concatenating) FC measures from all identified metrics, forming a composite multi-metric FC measure, i.e.,

18 $${\rho }_{composite}=({\rho }_{corr}|{\rho }_{xcorr}|{\rho }_{coh}|{\rho }_{wcoh}|{\rho }_{mutual\_info}|{\rho }_{euclidean}|{\rho }_{cityblock}|{\rho }_{dtw}|{\rho }_{emd})$$

### E3: Large-scale brain configurations based on functional connectivity

#### Objective

The large-scale brain networks, such as the DMN, ECN, language, etc., are typically derived from activation patterns from task functional MRI^6,8,43,48^. In experiments so far, these networks were assumed to be pre-defined and used to compare all FC measures. The goal of this experiment was to further characterize each FC measure by testing whether this assumption holds true across the alternative FC measures. In other words, the aim was to understand if FC patterns are dependent on the measure used to define it.

#### Participants

Data from only the young healthy group of 29 participants included in **E2** were considered here since these effects would first need to be tested and validated in a typical brain.

#### Behavioral data

In addition to neuroimaging, behavioral data were collected from these participants. Specifically, behavioral verbal fluency was measured by completing the following examination outside the scanner. Forms of the Controlled Oral Word Association Test (COWAT)^49^, were administered which requires participants to produce words beginning with the letters, “F,” “A,” “S” in three respective 1-min trials. Responses to each letter were recorded and verbal fluency scores were based on the total number of correct responses (after excluding perseverative and rule-breaking errors) produced by the participants across the three letter conditions. Since verbal fluency can be impacted by age and education, the raw scores were adjusted for each individual. The normalized verbal fluency score served as a behavioral outcome, most likely reflective of the language, working memory or executive control functions in the brain.

#### Data analysis

The analysis followed five main steps which are pictured in Supplementary Fig. 4. For each of the 9 FC measures:

(i) Ten large-scale brain networks consisting of 68 regions based on the atlas defined by Shirer *et al*.^8^, were included to generate a whole-brain symmetric FC matrix of size 68 × 68 for each individual. A mean FC matrix across all participants was used as a representative of group-level FC in subsequent steps. The idealized block structure along the diagonal of the FC matrix with 10 networks was defined as depicted in Supplementary Fig. 4. These matrices represent the *original brain configuration*.
(ii) The group mean FC matrix was shuffled by randomly permuting rows (and corresponding columns) so as to maintain the symmetry in FC matrix as an initialization step to remove potential bias of initial distribution on clustering result^50^. In the *original brain configuration*, the regions were arranged by network along the rows and columns symmetrically (i.e. regions in the same network appear in consecutive rows and columns). The shuffling process removes this network structure by randomly assigning each region to a different location while preserving the value of FC of each region to all others (i.e. symmetry).
(iii) A standard unsupervised *k*-means clustering algorithm was applied to this initialized, shuffled FC matrix with *k* = 10 (corresponding to the ten major brain networks that were part of the *original brain configuration*) and a sparsity (L1) distance function. Since the clustering by *k*-means algorithm is not unique, it was applied for 1000 iterations, each iteration initialized with a randomly shuffled FC matrix. An idealized block structure along the diagonal of the FC matrix with 10 clusters was defined for the clustered FC matrix as depicted in Supplementary Fig. 4. Clustered FC matrix and corresponding idealized FC matrix from each iteration represent an *alternative brain reconfiguration*.
(iv) Sørensen-Dice similarity coefficient was computed for the *original brain configuration* and each of the alternative *brain reconfigurations* by comparing the idealized FC structure and the thresholded FC matrix in each case. The two overlap coefficients were compared to determine the best possible functional configuration. The *ρ*_*composite*_ was also fed to the regression model for both configurations and compared.

To validate the plausibility of an *alternative brain reconfiguration*, brain-behavior associations were examined. A data-inspired linear regression was applied to study the association between the mean FC within a network (or cluster) of individual participants and their verbal fluency scores. In the *original brain configuration*, for each standard brain network, this procedure was performed to identify the specific FC measure that showed the best association with verbal fluency scores. In the best *alternative brain reconfiguration*, this was performed for each identified cluster. The strengths of this brain-behavior association were compared between the *original brain configuration* and the best observed *alternative brain reconfiguration*. Finally, a multi-metric approach was adopted similar to that in **E2**. For each FC measure, the mean FC within the one network was evaluated which exhibited the highest association with behavior, concatenated to form a composite multi-metric representation and then associated with the verbal fluency scores for the *original brain configuration*. Similarly, in the best *alternative brain reconfiguration*, the mean FC from the single cluster demonstrating the greatest association with behavior was computed for each FC measure, concatenated and associated with verbal fluency score. A stepwise regression model was employed which was initialized by including all linear terms of features (i.e., FC measures part of the composite multi-metric definition) and added/removed features with the criterion of maximizing the coefficient of determination (R^2^).

## Supplementary information

Supplementary Information.
