# Supplementary material for: Rethinking Measures of Functional Connectivity via Feature Extraction
Source: Sci Rep. 2020 Jan 28;10:1298. doi: 10.1038/s41598-020-57915-w (PMC6987226; doi:10.1038/s41598-020-57915-w)
Supplement: Supplementary file 1 — Supplementary Information. [file 41598_2020_57915_MOESM1_ESM.docx]

**Rethinking Measures of Functional Connectivity via Feature Extraction**

Rosaleena Mohanty^1,2,5*^, William A. Sethares^2^, Veena A. Nair^1^, Vivek Prabhakaran^1,3,4^

*^1^Department of Radiology, University of Wisconsin–Madison, Madison, WI, USA*

*^2^Electrical Engineering, University of Wisconsin-Madison, Madison, WI, USA*

*^3^Department of Medical Physics, University of Wisconsin–Madison, Madison, WI, USA*

*^4^Department of Psychiatry, University of Wisconsin–Madison, Madison, WI, USA*

*^5^Karolinska Institutet, Huddinge, Sweden*

**Supplementary Materials**

***Supplementary Figure 1.*** *Comparison of FC based on all measures in young healthy adults for* ***E1*** *in:* ***(a)*** *left motor* ***(b)*** *right motor and* ***(c)*** *language networks between task (sub-images* ***i****) and resting-state conditions (sub-images* ***ii****)*


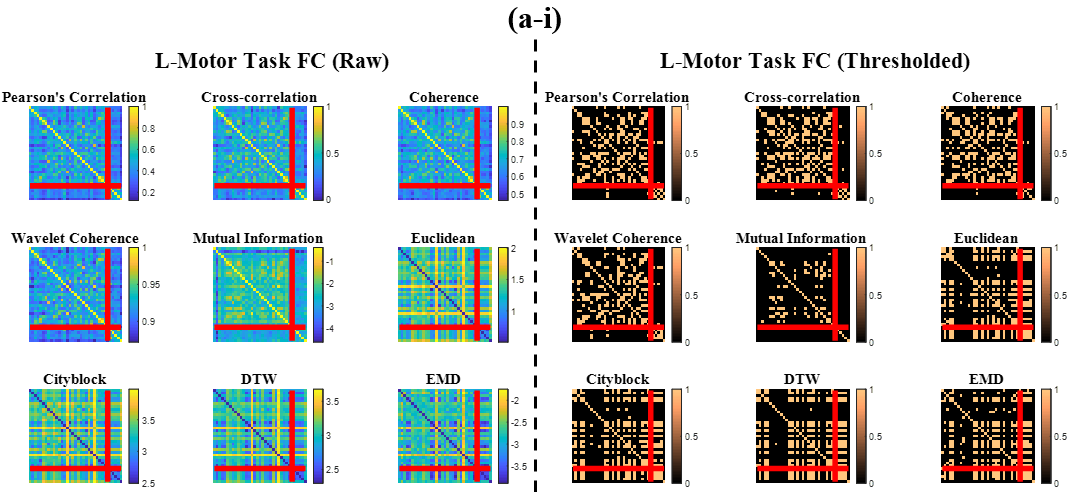


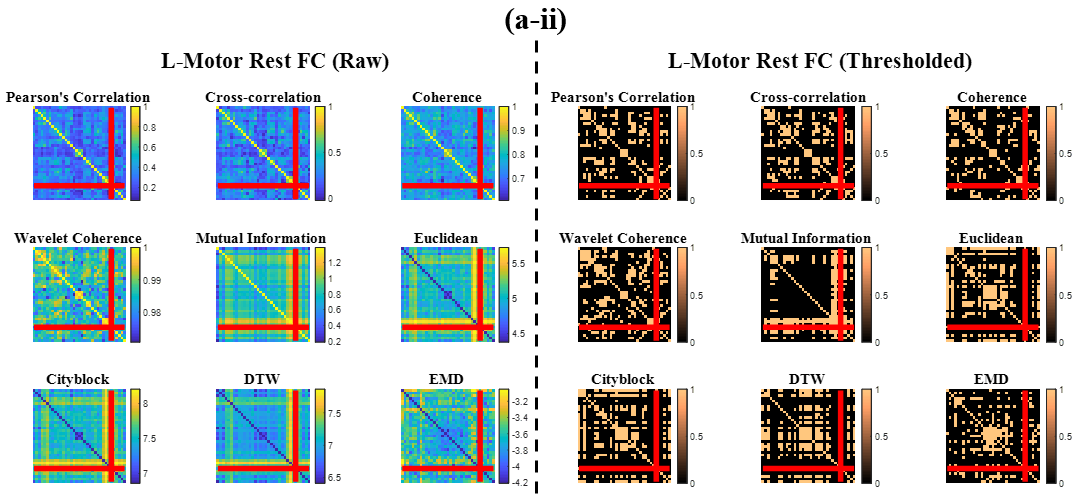


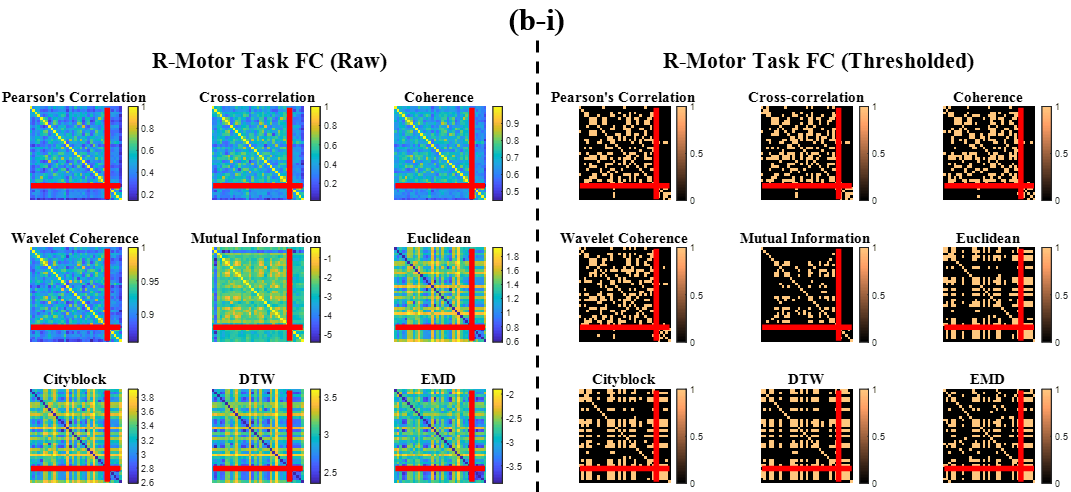


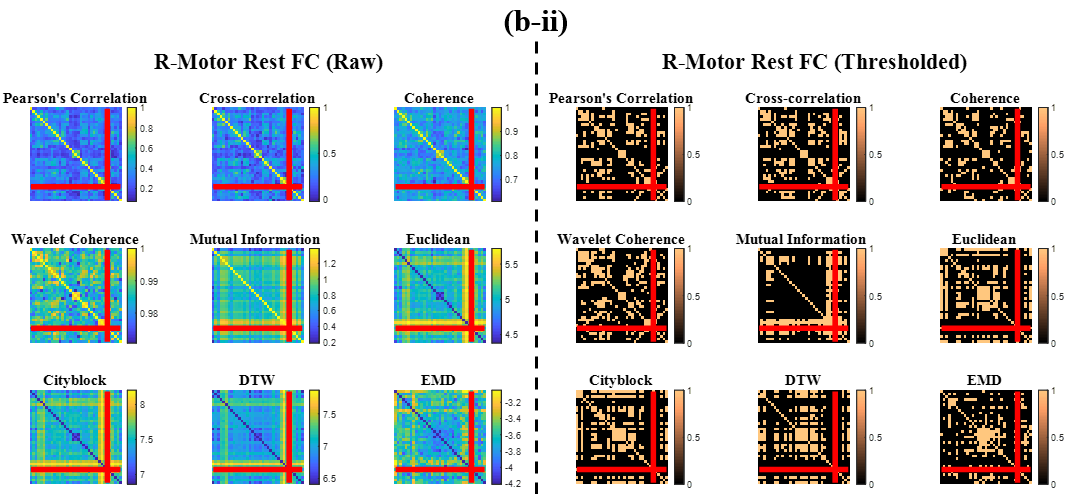


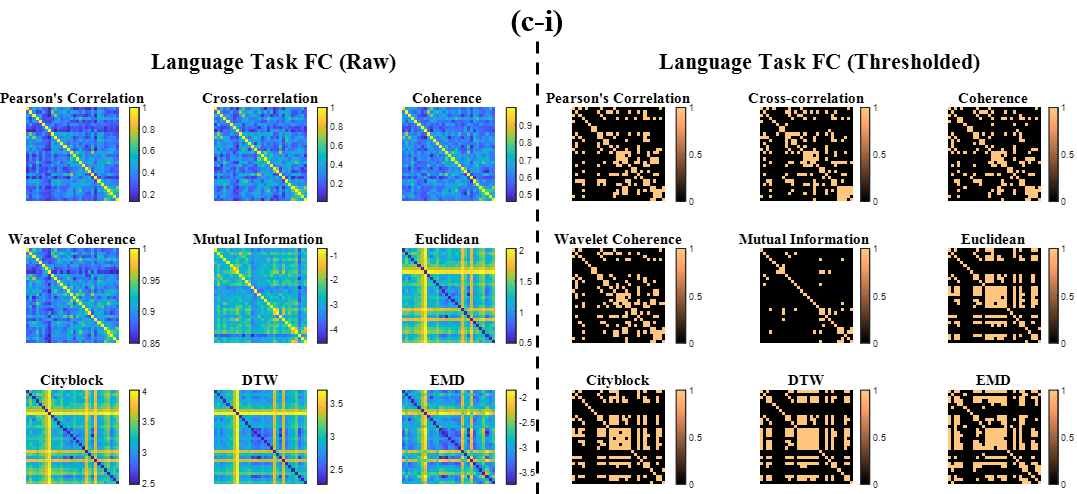


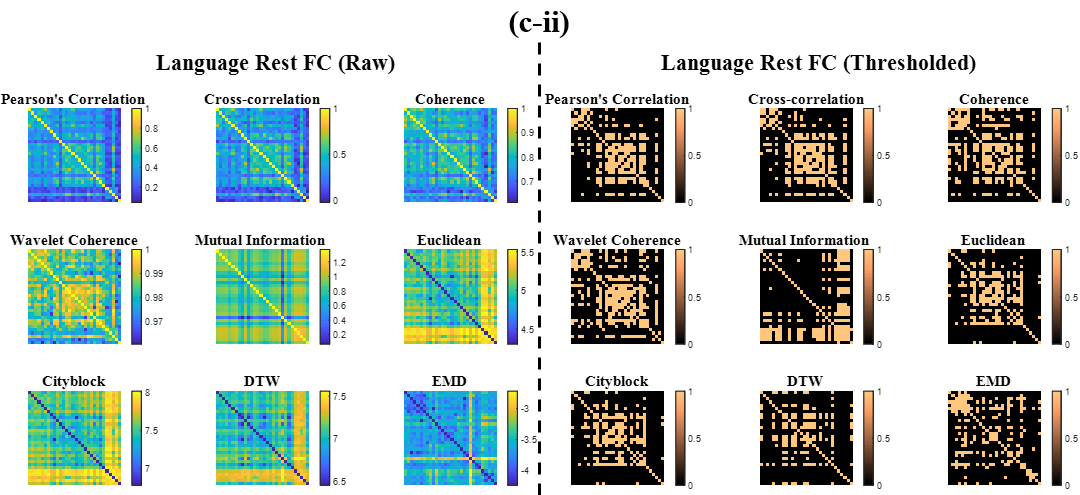


Note: Brain networks are defined based on Power functional atlas; In each image, matrices on the left represent FC averaged across all participants and those on right represent thresholded FC matrix averaged across all participants. The red lines in (a) and (b) show the separation between hand-motor and mouth-motor brain regions; DTW = dynamic time warp; EMD = Earth mover’s distance;

***Supplementary Figure 2.*** *Whole brain resting-state FC in young healthy adults defined based on various measures in 13 distinct brain networks in* ***E1****:* ***(a)*** *FC matrix averaged across all participants;* ***(b)*** *thresholded FC matrix averaged across all participants.*


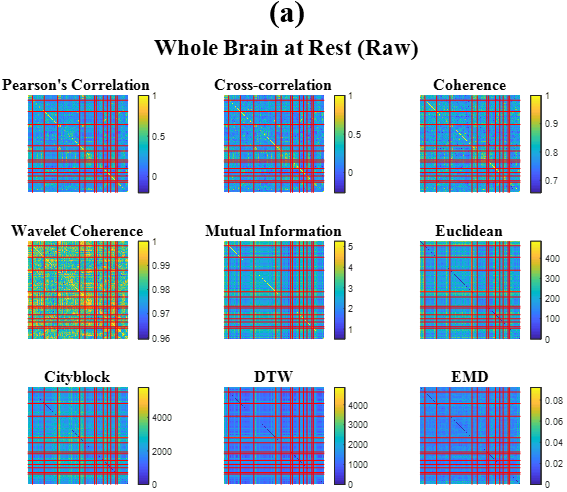


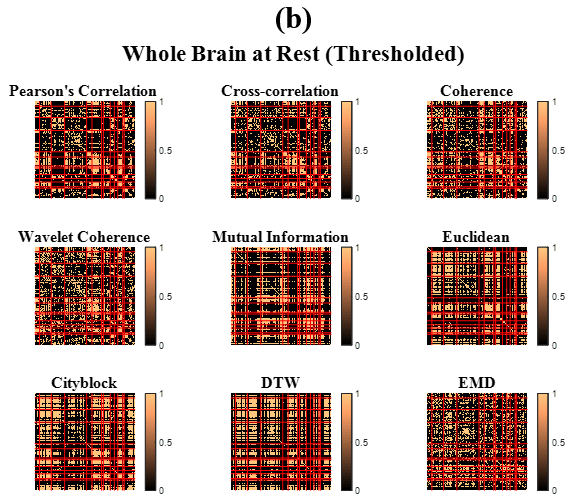


Note: Brain networks are defined on the basis of Power atlas. The red lines represent the separation between brain regions belonging to a specific network. The regions are grouped in the following order: audio, visual, motor, default mode, cingulo-opercular task, fronto-parietal task, memory, salience, dorsal attention, ventral attention, subcortical, cerebellar, uncertain networks; DTW = dynamic time warp; EMD = Earth mover’s distance;

***Supplementary Figure 3.*** *The distribution of Sørensen-Dice similarity coefficients obtained for 1000 iterations of k-means clustering.*


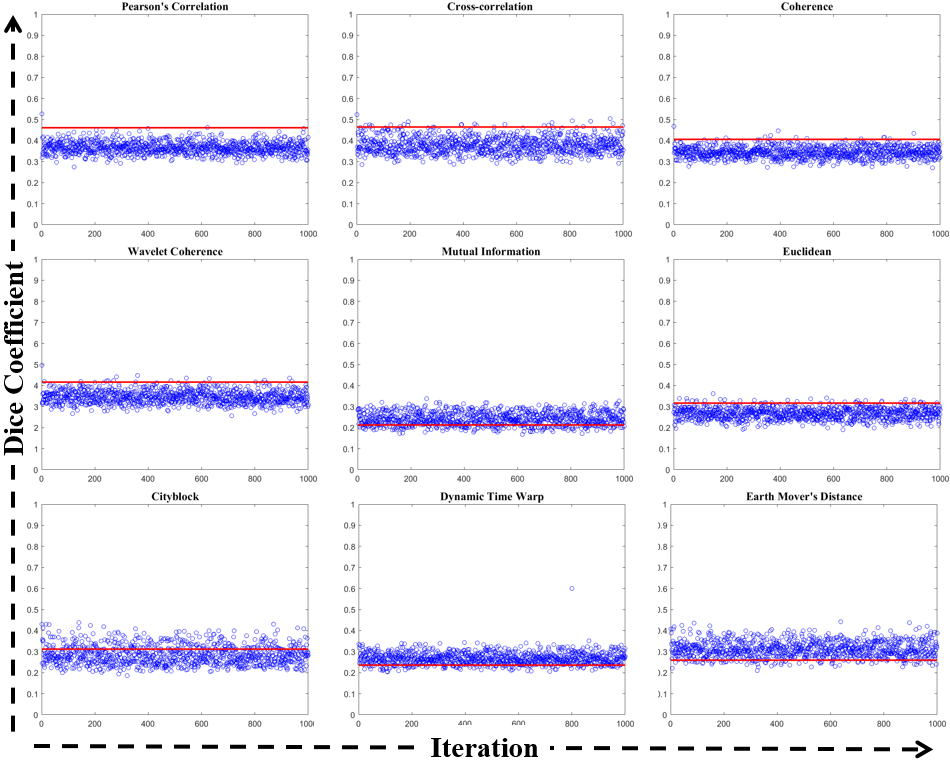


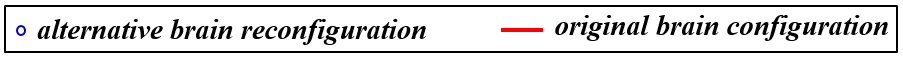


Note: The red line represents the Sørensen-Dice similarity coefficient achieved in the *original brain configuration* whereas the Sørensen-Dice similarity coefficient obtained for each of the *alternative brain reconfigurations* is represented by a blue circle.

***Supplementary Figure 4.*** *The pipeline designed for* ***E3*** *(shown for Pearson’s correlation)*


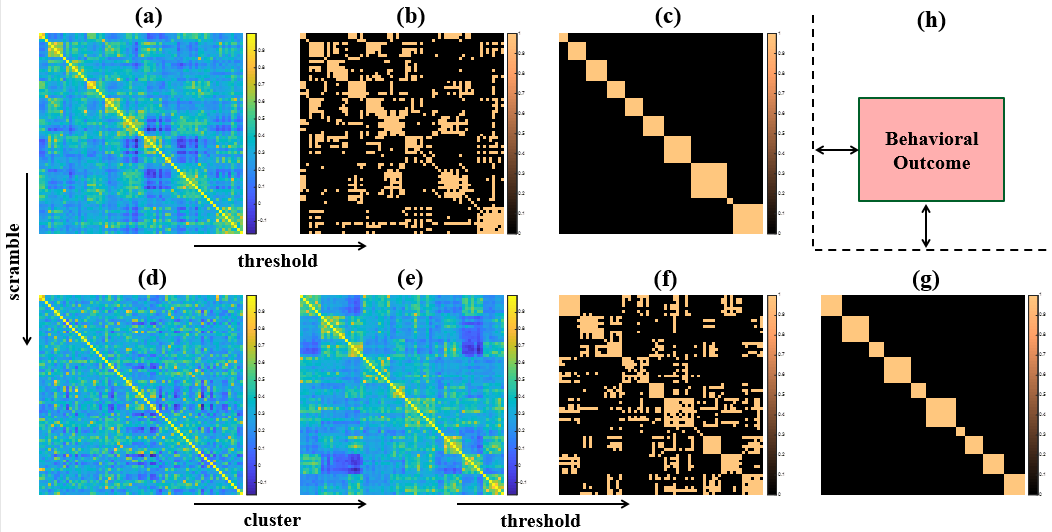


Note: **(a)** *original brain configuration* consisting of 68 regions and 10 large-scale brain networks averaged over FC matrices of 29 young healthy adults; **(b)** thresholded version of *original brain configuration*; **(c)** an ideal block structure corresponding to the *original brain configuration*; **(d)** a random scrambling of the *original brain configuration* as an initialization step for clustering; **(e)** clustered FC matrix based on *k*-means with *k*=10 and a sparsity-based distance function resulting in an *alternative* *brain reconfiguration*; **(f)** thresholded version of *alternative* *brain reconfiguration*; **(g)** the ideal clock structure corresponding to the *alternative* *brain reconfiguration*; Sørensen-Dice similarity coefficient for the *original brain configuration* and the *alternative* *brain reconfiguration* are compared to find whether the latter is a better arrangement; **(h)** plausibility of reconfigured arrangement is validated by associating FC from **(g)** with behavioral outcome, i.e. normalized verbal fluency score and compared to association between **(c)** with behavioral outcome.

***Supplementary Figure 5.*** *Comparison of the* ***(a)*** *original brain configuration and* ***(b)*** *alternative brain reconfiguration for FC measures which achieved the best Sørensen-Dice similarity coefficient (****Table 5****).*


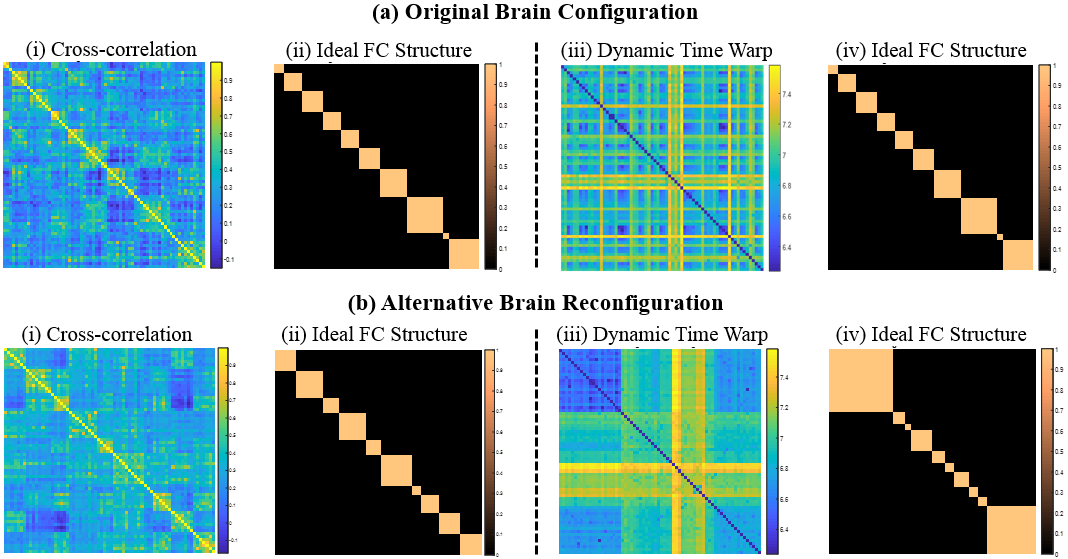


Note: For each (a) and (b): (i) whole brain FC matrix (comprised of 10 networks) for cross-correlation; (ii) ideal structure of FC matrix corresponding to the configuration in (i); (iii) whole brain FC matrix (comprised of 10 networks) for dynamic time warp; (iv) ideal structure of FC matrix corresponding to the configuration in (i);

***Supplementary Table 1.*** *Age-based classification between younger and older healthy adults in nine major brain networks in* ***E2****:* ***(A)*** *Area under the curve for individual classifier*

| **FC Measure** | **D. DMN** | **V. DMN** | **L. ECN** | **R. ECN** | **A. Sal.** | **P. Sal.** | **Auditory** | **Language** | **Motor** |
| --- | --- | --- | --- | --- | --- | --- | --- | --- | --- |
| Pearson’s Correlation | 0.42 | 0.50 | 0.38 | 0.61 | 0.83 | 0.63 | 0.65 | 0.55 | 0.51 |
| Cross-correlation | 0.50 | 0.50 | 0.46 | 0.65 | 0.82 | 0.60 | 0.56 | 0.47 | 0.56 |
| Coherence | 0.59 | 0.50 | 0.52 | 0.51 | 0.61 | 0.60 | 0.53 | 0.52 | 0.65 |
| Wavelet coherence | 0.36 | 0.47 | 0.55 | 0.57 | 0.70 | 0.45 | **0.69** | 0.58 | 0.26 |
| Mutual Information | 0.73 | 0.71 | 0.66 | 0.55 | 0.77 | 0.55 | 0.52 | 0.57 | 0.61 |
| Euclidean | 0.67 | **0.73** | 0.71 | **0.68** | 0.82 | 0.53 | 0.47 | 0.55 | 0.49 |
| Cityblock | 0.70 | 0.74 | 0.70 | **0.69** | 0.83 | 0.49 | 0.51 | 0.63 | 0.52 |
| DTW | **0.79** | 0.72 | **0.77** | 0.66 | 0.83 | 0.59 | 0.50 | **0.66** | 0.36 |
| EMD | 0.47 | 0.65 | 0.66 | 0.55 | 0.68 | 0.58 | 0.56 | 0.45 | **0.67** |
| Composite | 0.74 | 0.64 | 0.75 | 0.53 | **0.91** | **0.69** | 0.68 | 0.53 | 0.54 |

Note: Brain networks are defined by Willard functional atlas; classification is performed with a support vector machine classifier; performance represents area under the curve with a leave-one out testing; the highest performing FC measure is represented in **bold** for each network; D.DMN = dorsal default mode network; V.DMN = ventral default mode network; L.ECN = left executive control network; R.ECN = right executive control network; A.Sal. = anterior salience; P.Sal. = posterior salience; DTW = dynamic time warping; EMD = Earth mover’s distance;

***Supplementary Table 1.*** *Age-based classification between younger and older healthy adults in nine major brain networks in* ***E2****:* ***(B)*** *The minimum and maximum number of features selected in each classification model in the format [minimum, maximum]*

| **FC Measure** | **D. DMN** | **V. DMN** | **L. ECN** | **R. ECN** | **A. Salience** | **P. Salience** | **Auditory** | **Language** | **Motor** |
| --- | --- | --- | --- | --- | --- | --- | --- | --- | --- |
| Pearson’s Correlation | [3, 7] | [4, 9] | [3, 8] | [2, 6] | [1, 3] | [4, 8] | [1, 1] | [3, 21] | [3, 5] |
| Cross-correlation | [1, 7] | [3, 9] | [2, 6] | [1, 4] | [1, 3] | [3, 8] | [3, 3] | [1, 21] | [1, 4] |
| Coherence | [3, 10] | [3, 8] | [2, 4] | [1, 15] | [3, 5] | [3, 11] | [1, 1] | [1, 7] | [1, 1] |
| Wavelet coherence | [1, 4] | [1, 5] | [2, 4] | [1, 2] | [1, 2] | [1, 5] | [1, 2] | [1, 2] | [1, 15] |
| Mutual Information | [1, 3] | [1, 3] | [2, 3] | [2, 3] | [1, 2] | [1, 4] | [3, 3] | [1, 1] | [1, 2] |
| Euclidean distance | [3, 5] | [1, 3] | [1, 3] | [1, 1] | [2, 3] | [2, 4] | [3, 3] | [1, 3] | [1, 4] |
| Cityblock distance | [3.4] | [1, 3] | [1, 4] | [1, 2] | [2, 3] | [3, 7] | [3, 3] | [2, 4] | [1, 4] |
| DTW | [3, 6] | [1, 4] | [1, 2] | [1, 2] | [1, 2] | [2, 5] | [1, 3] | [1, 2] | [1, 2] |
| EMD | [1, 36] | [5, 8] | [2, 5] | [1, 15] | [2, 2] | [3, 11] | [3, 3] | [2, 5] | [1, 3] |
| Combined | [7, 15] | [5, 17] | [5, 13] | [5, 14] | [5, 8] | [9, 20] | [3, 5] | [5, 17] | [3, 9] |

Note: Brain networks are defined by Willard functional atlas; classification is performed with a support vector machine classifier; D.DMN = dorsal default mode network; V.DMN = ventral default mode network; L.ECN = left executive control network; R.ECN = right executive control network; A.Sal. = anterior salience; P.Sal. = posterior salience; DTW = dynamic time warping; EMD = Earth mover’s distance;

***Supplementary Table 2.*** *Multiple measures used to characterize FC and their properties*

| **Measure** | **Properties** | **Implementation (MATLAB)** |
| --- | --- | --- |
| Pearson’s Correlation | - Time-domain - Similarity - Linear - Scale-invariant | - ‘corr’ function |
| Cross-correlation | - Time-domain - Similarity - Linear - Scale-invariant | - ‘xcorr’ function - maximum value across all possible shifts was used to measure FC |
| Coherence | - Frequency-domain - Similarity - Linear - Scale-invariant | - ‘mscohere’ function - maximum value across all possible frequencies was used to measure FC |
| Wavelet coherence | - Time-frequency-domain - Similarity - Linear - Scale-invariant | - ‘wcoherence’ function - average value over the instances (top half only) showing highest similarity was used to measure FC |
| Mutual information | - Time-domain - Similarity - Non-linear - Scale-variant | - ‘mutualinfo’ function - package used: [Mathworks File Exchange mutualinfo](https://www.mathworks.com/matlabcentral/fileexchange/14888-mutual-information-computation?focused=5090031&tab=function) |
| Euclidean distance | - Time-domain - Dissimilarity - Linear - Scale-variant | - ‘pdist’ function - Distance = ‘euclidean’ |
| Cityblock distance | - Time-domain - Dissimilarity - Linear - Scale-variant | - ‘pdist’ function - Distance = ‘cityblock’ |
| Dynamic time warping | - Time-domain - Dissimilarity - Non-linear - Scale-variant | - ‘dtw’ function - Metric = ‘euclidean’ |
| Earth mover’s distance | - Time-domain - Dissimilarity - Non-linear - Scale-variant | - ‘emd’ function - nbins = 10 - package used: [MathWorks File Exchange emd](https://www.mathworks.com/matlabcentral/fileexchange/22962-the-earth-mover-s-distance) |
